# Adipose Tissue, Obesity and Adiponectin: Role in Endocrine Cancer Risk

**DOI:** 10.3390/ijms20122863

**Published:** 2019-06-12

**Authors:** Andrea Tumminia, Federica Vinciguerra, Miriam Parisi, Marco Graziano, Laura Sciacca, Roberto Baratta, Lucia Frittitta

**Affiliations:** Endocrinology, Department of Clinical and Experimental Medicine, University of Catania, Garibaldi Hospital, Via Palermo 636, 95122 Catania, Italy; andreatumminia@libero.it (A.T.); vinciguerrafederica@gmail.com (F.V.); mrmparisi@gmail.com (M.P.); graziano.marco91@gmail.com (M.G.); lsciacca@unict.it (L.S.); rob.baratta@gmail.com (R.B.)

**Keywords:** adiponectin, adipose tissue, obesity, endocrine cancer

## Abstract

Adipose tissue has been recognized as a complex organ with endocrine and metabolic roles. The excess of fat mass, as occurs during overweight and obesity states, alters the regulation of adipose tissue, contributing to the development of obesity-related disorders. In this regard, many epidemiological studies shown an association between obesity and numerous types of malignancies, comprising those linked to the endocrine system (e.g., breast, endometrial, ovarian, thyroid and prostate cancers). Multiple factors may contribute to this phenomenon, such as hyperinsulinemia, dyslipidemia, oxidative stress, inflammation, abnormal adipokines secretion and metabolism. Among adipokines, growing interest has been placed in recent years on adiponectin (APN) and on its role in carcinogenesis. APN is secreted by adipose tissue and exerts both anti-inflammatory and anti-proliferative actions. It has been demonstrated that APN is drastically decreased in obese individuals and that it can play a crucial role in tumor growth. Although literature data on the impact of APN on carcinogenesis are sometimes conflicting, the most accredited hypothesis is that it has a protective action, preventing cancer development and progression. The aim of the present review is to summarize the currently available evidence on the involvement of APN and its signaling in the etiology of cancer, focusing on endocrine malignancies.

## 1. Introduction

Obesity represents a condition of chronic excess fat mass. Several epidemiological studies have revealed an alarming increase in the number of obese individuals worldwide [1]. It is important to emphasize that obesity represents a risk factor for the onset of different metabolic disorders, such as type 2 diabetes, as well as for the development of cardiovascular diseases [2]. Moreover, it has been well established that the risk of many types of malignancies is increased in obese individuals [3]. Recent evidence indicates, indeed, that excess adiposity is associated with about 20% of all cancers [4]. For these reasons, obesity is a substantial public health challenge, representing one of the major causes of avoidable mortality and morbidity [5]. 

Molecular mechanisms linking excessive adiposity with the development of cancer are complex and still not completely known. Multiple factors potentially contribute to this relationship. Obesity is, in fact, often related to metabolic defects that may favor not only cancer initiation, but also its progression [6]. These abnormalities include: adipose tissue low-grade inflammation, which implies the production of specific inflammatory adipocytokines, oxidative stress, peripheral insulin resistance with hyperinsulinemia and dyslipidemia [7,8]. In particular, growing interest has been recently placed on the role of adipose tissue-secreted molecules in the development of cancer [9]. Adipose tissue, initially thought as a mere fat mass depot, is now widely recognized as an active endocrine organ [10]. It secretes different types of molecules called adipokines, which are implicated in the pathogenesis of numerous types of malignancies [9,11]. Among others, adiponectin (APN) has been demonstrated to have several functions in human physiology balancing glucose and lipid metabolism and revealing insulin-sensitizing, anti-apoptotic and immune regulatory effects [10,12,13]. Hypoadiponectinemia has been, in fact, consistently associated with obesity-related insulin resistance and type 2 diabetes, as well as with a higher risk of various cancer types [9,14], and thus this molecule has generally been considered a beneficial adipokine. Indeed, several studies have demonstrated that increasing plasma APN levels and, therefore, the activation of its intracellular signaling, are able to mitigate the deleterious effects of metabolic dysfunctions on tumor development and progression [15]. Thus, the possibility of mimicking some of the cancer-protective properties of APN has attracted significant interest within the scientific community for the potential therapeutic applications of this approach. However, research on the role of APN on tumor growth has provided evidence for both positive and negative influences, raising doubts on the previously thought protective role of APN on cancer risk and progression [16]. Even more unexpected were data on the role of APN on the risk of all-cause mortality. In fact, a positive, rather than negative, relationship has been reported between APN and death rates across various clinical conditions, including different types of malignancy [17]. Therefore, understanding the complexity of APN’s metabolism, and linking its signaling pathway to cancer development and prognosis, represent a challenging task. 

In this review we will summarize the currently available data on this topic, mainly focusing on endocrine malignancies (e.g., breast, endometrial, ovarian, thyroid and prostate cancers), which seem to be deeply linked to dysfunctional APN secretion and action. 

## 2. APN Structure and Receptors 

**Figure 1:**
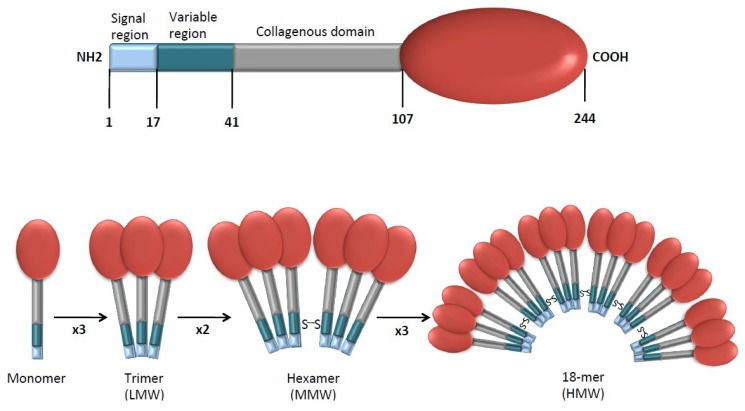
APN’s molecular structure and isoforms. Monomeric APN is able to trimerize to form low molecular weight (LMW) APN. Two trimers can then combine to form middle molecular weight (MMW) hexamers. The trimers are able to form 12- or 18-mers with high molecular weight (HMW).

APN is encoded by AdipoQ, a gene that makes a monomeric molecule made up of 244 amino acids and consists of a signal region at the NH2-terminus, a variable region, a collagenous domain and a globular domain at the COOH-terminus (Figure 1) [18]. 

After post-translational modifications, APN circulates in trimeric, hexameric, and multimeric high-molecular weight (HMW) isoforms. Each isoform is able to activate distinct signal transduction pathways, regulating various biological functions [10]. Moreover, a globular version of APN, resulting from proteolytic cleavage of the COOH-terminal domain, circulates in small concentrations in plasma. 

The different APN isoforms mediate distinct effects in various tissues and organs. For example, the HMW isoform, which is the most biologically active, is believed to mediate the pro-inflammatory effects of APN, whereas the trimeric isoform has been suggested as responsible for its anti-inflammatory activity [19]. 

APN acts through its classical receptors, AdipoR1 and AdipoR2, which have been demonstrated to have different binding affinity for the different APN isoforms [20]. There are seven trans-membrane domain receptors that activate a signaling cascade, leading to numerous metabolic and immune-related effects. AdipoR1 is expressed almost ubiquitously, whereas AdipoR2 is mostly expressed in hepatocytes and white adipose tissue [20]. In addition, a non-classical APN receptor, the T-Cadherin (which acts through calcium dependent mechanisms) has been found to bind the hexameric and HMW species of APN, but not the trimeric or globular species [21].

## 3. Adiponectin Signaling and Mechanisms of Carcinogenesis

The role of APN in endocrine cancer risk is deeply linked to many complex dysfunctions, including an altered adipose tissue homeostasis and the activation of multiple epigenetic pathways within tumor cells and neoplastic microenvironment [22]. A correlation between hypoadiponectinemia, obesity and hormonally influenced cancers has been found in several clinical studies [23,24]. While the majority of evidence shows an inverse correlation between APN and endocrine malignancies, another group of studies associates increased circulating APN levels with tumor progression [25]. Indeed, it has been proposed that low APN concentrations could be associated with cancers linked to an excess of fat mass and to sex steroid hormones, while higher plasma APN levels might indicate high levels of inflammation and advanced stages of malignancy [25,26]. 

A robust amount of studies in the past two decades have suggested that APN exerts its antineoplastic effects on endocrine cancers via two main mechanisms. First, it can affect endocrine tumor growth by acting directly on cancer cells through receptor-mediated pathways. Secondly, it may indirectly influence cancer biology by modulating insulin sensitivity, inflammation and tumor angiogenesis [23]. In the following section we will briefly discuss the in vitro and in vivo observations of these different mechanisms.

### 3.1. Direct Mechanisms: Receptor-Mediated and Paracrine

**Figure 2:**
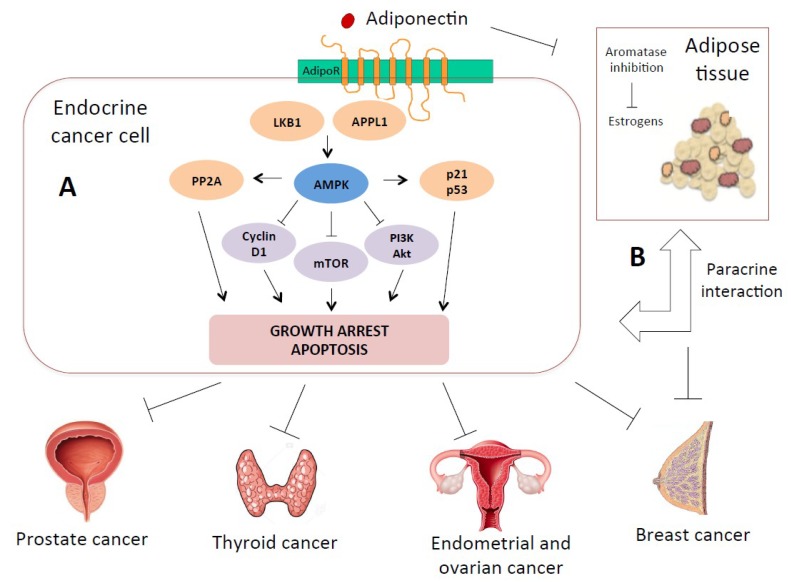
APN’s receptor-mediated and paracrine actions on endocrine cancer cells. (**A**) APN activates adenosine monophosphate-activated protein kinase (AMPK) via an increased expression of the adaptor protein APPL-1 as well as the Ser/Thr kinase LKB1. AMPK activation affects cell growth by inducing p53, p21 and phosphatase 2A (PP2A) expression. Down-regulation of the mammalian target of rapamycin (mTOR), PI3K/Akt and Cyclin D1 signaling is also implicated in the APN-mediated growth arrest and apoptosis; (**B**) In adipocytes, APN inhibits aromatase activity, lowering estrogen production and reducing ERα-stimulation in adjacent breast cancer cells. It negatively affects pro-survival pathways.

The main direct mechanism determining APN’s protective role on endocrine cancer cells is the activation of adenosine monophosphate-activated protein kinase (AMPK). This protein represents a crucial regulator of energy balance, as it is responsible for cellular adaptation to metabolically challenging states such as inflammation and oxidative stress. In such conditions, AMPK turns off anabolic and proliferative pathways, while increasing the production of adenosine triphosphate (ATP) [27]. APN stimulates AMPK through an increase of AMP levels, and by means of various cellular mediators comprising the adaptor protein APPL-1, calcium-dependent kinases and the liver kinase B1 (LKB1) [28]. Specifically, it has been demonstrated that LKB1 plays a fundamental role in necessitating breast cancer cells for AMPK activation, and for the consequent inhibition of tumor cell adhesion, migration and invasiveness [29]. AMPK activation negatively influences cancer development by affecting some of the key mechanisms that regulate cell growth [30]. In fact, it is able to induce the expression of important molecules involved in cell cycle arrest and apoptosis, such as p53 and p21 [31]. This mechanism of action has been proven in many types of malignancies, especially endocrine cancers. APN treatment was in fact shown to stimulate AMPK in breast [32,33], prostate [34,35] and endometrial cancers [36], mediating tumor growth inhibition. Down-regulation of the mammalian target of rapamycin (mTOR) signaling pathway was implicated in many of these studies [29,34]. Moreover, in MDA-MB-231 breast cancer cells, APN-related AMPK pathway activation has been demonstrated to induce protein phosphatase 2A (PP2A), a tumor suppressor protein involved in Akt dephosphorylation [37,38]. Additionally, some studies have shown a direct negative influence of APN on the PI3K/Akt signaling pathway, which determines a series of events leading to cell death and, therefore, to tumor growth inhibition (Figure 2) [11].

APN also modulates signal transducer and activator of transcription 3 (STAT3) signaling. STAT3 is activated by adipokine-induced JAK phosphorylation and regulates various processes related to cancer development and progression [39]. For example, it has been demonstrated that APN treatment is able to down-regulate leptin-induced STAT3 phosphorylation, reducing tumor cell growth [39]. Furthermore, APN has been shown to regulate the cAMP/PKA pathway, modulating anti-proliferative actions leading to cell apoptosis in MCF7 breast cancer cells [32].

Of note, APN influence in endocrine cancer cells may also depend on paracrine interactions between adipocytes and tumor cells, being that these cell types are in close proximity to each other [40]. A typical example of this mechanism can be seen in the case of breast cancer [40]. In that situation, APN determines an inhibition of aromatase activity in adipocytes, lowering estrogen production and reducing estrogen receptor alpha (ERα) stimulation in adjacent breast cancer cells. This phenomenon negatively affects pro-survival pathways [40,41]. However, it is important to emphasize that the molecular links between adipose tissue and endocrine cancer cells are far more complex and not yet fully characterized. They involve, in fact, other adipocyte-secreted molecules (e.g., leptin and resistin), numerous inflammatory cytokines (e.g., TNFα, IL-6), extracellular matrix elements, pro-angiogenic factors (e.g., vascular endothelial growth factor (VEGF)), as well as metabolic regulators like insulin and insulin-like growth factor I (IGF-I) [40,42].

### 3.2. Indirect Mechanisms: Insulin-Sensitizing, Immune-Related, Anti-Angiogenic Effects 

Through different mechanisms, APN can exert indirect antineoplastic actions, including insulin-sensitizing, immune-related and angiogenesis-related effects, although conflicting evidence has been published [23]. An indirect link between the insulin pathway, AP, and endocrine carcinogenesis has been shown in many studies. It is well documented that insulin supports tumor cell proliferation [43,44]. Serum APN levels appear to be inversely related to fasting insulin concentrations, and are reduced in conditions of insulin resistance [45]. Since these metabolic conditions represent risk factors for endocrine cancer development, APN might act as an anticancer agent due to its significant effect on insulin post-receptor signaling [46]. Specifically, APN is a strong inhibitor of the PI3K/Akt/mTOR pathway, being able to reduce tumor cell growth induced by insulin and by other growth factors [47].

Immune system deregulation represents a crucial pathophysiological factor in determining increased cancer risk [48,49]. Abnormal immune response is an important constituent of obesity, and contributes to the development of obesity-related disorders such as cancer. Adipose tissue overgrowth accompanies the infiltration of various types of immune cells from both innate and adaptive immunity [50]. Immune cells infiltrating tumor microenvironments and adipocytes secrete pro-inflammatory adipocytokines, providing a condition of low-grade inflammation which favors cancer initiation and progression [51]. Specifically, macrophage infiltration and its phenotypic switching toward an M1 phenotype, constitute critical mechanisms related to increased tumor growth in settings of excess adiposity [52]. In pathological conditions characterized by a chronic inflammatory response such as an infection, but also metabolic diseases such as obesity, type 2 diabetes and atherosclerosis, a lowering of serum APN concentrations has been observed [53]. Obesity, in particular, is associated with increased pro-inflammatory markers such as IL-6, TNF-α and c-reactive protein (CRP). APN has been shown to exert immune regulatory and anti-inflammatory actions and may thus mitigate the increased risk of cancer development related to states of obesity-induced inflammation [54]. Particularly, APN influences the function of myelomonocytic cells that are important regulators of innate immunity. Moreover, APN negatively affects macrophage phagocytic activity [55].

One of the main mechanisms of cancer development is the recruitment of blood vessels to provide nutrients and oxygen to cancer cells [56]. Inhibition of angiogenesis has been demonstrated to suppress tumor growth and may therefore represent a promising therapeutic option [57,58]. Regarding the role of APN in angiogenesis and cellular proliferation, the literature appears to be contradictory. Several studies suggest, in fact, that APN has an anti-angiogenic effect both in vitro and in vivo. In particular, Brakenhielm et al. characterized the strong inhibition of angiogenesis exerted by APN [57], which involves specific signaling pathways such as the mitogen-activated protein kinase (MAPK) and cAMP-PKA pathways [59]. Conversely, convincing molecular data suggest that APN might have a powerful pro-angiogenic effect that could promote cancer development particularly in murine mammary tumor models [60]. Further studies are needed to better define the role of APN on tumor angiogenesis.

## 4. Breast Cancer 

Breast cancer represents the most common malignancy in the female sex and the second most frequent tumor worldwide [61]. In both premenopausal and postmenopausal patients, obesity is considered an important risk factor for the development and progression of breast cancer, lowering a patient’s chances of survival [62].

Several studies have investigated the role of adipocytokines in breast cancer, suggesting a pivotal role of APN in its development and recurrence [63,64]. Within the mammary gland, epithelial cells included in peri-glandular adipose tissue are exposed to both circulating and locally secreted adipokines from adjacent adipocytes. As mentioned above, this paracrine interaction, in addition to the circulating effect of the hormone, may influence breast cancer development [65,66]. 

APN demonstrated in vitro anti-proliferative and pro-apoptotic effects on breast cancer cells, suppressing cell growth and proliferation, and inhibiting the migration and invasion capabilities of cancer cells [33,67,68,69]. Moreover, epidemiological studies reported a significant inverse association between APN and breast cancer risk (Table 1) [70,71,72]. This association appears to be stronger for postmenopausal women, although contrasting data have been sometimes published [73]. Significantly lower APN levels have been shown in women with breast cancer compared to healthy controls, especially during the postmenopausal period, suggesting that APN might influence proliferation of breast cancer cells in a low estrogen environment [70,74,75,76]. Conversely, other studies have indicated a stronger association in women of childbearing age [33,77]. A randomized trial conducted on premenopausal women with intraepithelial neoplasia or micro-invasive breast cancer demonstrated that baseline APN levels predict new breast events. After a median of 7.2 years, a 12% reduction in the risk of developing breast cancer was reported per unit increase of APN [78]. Recently, a large meta-analysis of 31 studies concluded that low serum APN concentrations might be linked to an increased risk of breast cancer in female patients regardless of age [79]. An inverse association between plasma APN levels and increased tumor aggressiveness has also been shown: low APN concentrations were associated with larger tumors, higher histological grade and increased metastasis rate [70,74,80]. Moreover, it has been reported that lower plasma APN concentrations represent a risk factor for progression from intraepithelial to invasive cancer, regardless of age or body mass index (BMI) [78]. Another recent meta-analysis including 27 case–control studies confirmed that serum APN might be inversely associated with breast cancer, but suggested differences in ethnicity, showing higher associations in Asian than Caucasian women [81]. These differences across ethnicity have not been reported in other previously published meta-analyses [73,82]. Authors speculate that several factors influencing serum APN concentrations, like different lifestyle and dietary habits or fat distribution, may explain these results [81,83]. 

The mechanisms through which APN determines its protective role against breast cancer are yet unclear, but various molecular mechanisms have been proposed [79]. Specifically, it is known that low serum APN levels are associated with hyperinsulinemia, which has been demonstrated to promote the proliferation of tumor cells acting as a growth factor through insulin and IGF-I receptors [84,85]. Furthermore, Brakenhielm et al. demonstrated that APN acts as a negative modulator of angiogenesis by suppressing endothelial cell proliferation [57]. It also induces a signaling cascade that results in apoptosis through the activation of caspases 3, 8 and 9 [57]. APN may also affect breast cancer risk by altering serum estrogen levels; it has, in fact, been demonstrated that APN levels are negatively associated with estrogen concentrations [86]. While these data contribute to understanding the crucial role of APN on breast cancer pathophysiology, several studies have not confirmed these findings [75,76,77,87]. According to some authors [88], this discrepancy could be partially due to the presence of various APN isoforms with different molecular weights. In particular, an increased breast cancer risk has been specifically related to low levels of the HMW isoform, rather than to total APN [88]. 

It has been also suggested that APN’s effect on breast cancer growth may differ in relation to ERα expression. Most studies show that in ERα-negative breast cancer cells, APN has an anti-proliferative and pro-apoptotic effect [89,90,91]. Instead, ERα positivity seems to negatively interfere with the anti-proliferative effect of APN on breast cancer cell growth [92,93]. In ERα-positive cells, low APN levels favor the interaction of APPL1 (a mediator of signaling pathways of cell proliferation, apoptosis and cell survival) with AdipoR1, ERα, IGF-IR and c-Src, determining MAPK phosphorylation. This interaction promotes ERα activation at genomic levels, inducing breast cancer cell proliferation [92]. In contrast, APN-induced AMPK/LKB1 pathway activation results in mTOR inhibition in ERα-negative cells, limiting breast cancer progression [94]. Different genes appear to be involved in the anti-proliferative action of APN in ERα-negative breast cancer cells. They include p53, Bax, Bcl-2, c-myc and cyclin D1 [67,90,95]. Therefore, APN is able to inhibit ERα-negative cell growth and progression both in vitro and in vivo.

Despite the complexity of the association between APN and breast cancer, the preponderance of evidence suggests a correlation between low serum PN concentrations and breast cancer risk (Table 1). Further large case–control studies are necessary to better explain the role of APN on the different breast cancer phenotypes and among different ethnicities.

## 5. Endometrial Cancer

Endometrial cancer is the most frequent gynecologic malignancy and the fourth most common type of cancer among women. It has a worldwide incidence of about 280,000 new cases annually [111].

Obesity represents one of the most important risk factors for the onset of endometrial cancer [112]. An excess of fat mass leads to reduced serum levels of sex hormone binding globulin (SHBG) and progesterone, which results in an increased amount of bioavailable testosterone and estrogen. These hormonal alterations constitute a stimulus for the proliferation of the endometrium and, therefore, for the development of endometrial cancer. It has been estimated that patients with first-degree obesity (BMI 30–35 kg/m^2^) or severe obesity (BMI > 35 Kg/m^2^) have respectively a 2.5 times and 5 times higher risk of developing endometrial cancer than average-weight patients [113]. This correlation appears to be even more critical because it has been recently reported that pre-pubertal obesity (7–13 years) is associated with the onset of this type of cancer in adult age [114]. Moreover, several studies correlate a patient’s weight loss with a reduction of endometrial cancer risk [16].

The excess of adipose tissue may exacerbate endometrial cancer risk through several other mechanisms, including insulin resistance, excessive aromatization of adrenal androgens in the adipocytes (resulting in higher levels of endogenous estrogens), chronic inflammation and the production of several adipokines (including APN) [115]. In a recent meta-analysis, low APN levels, typical of obese women, were associated with a 53% greater risk of developing endometrial cancer [99]. This inverse correlation between plasma APN concentration and endometrial cancer risk is supported by most of the data published on this topic [96,100,116,117]. In particular, a strong inverse association was demonstrated in peri- or post-menopausal patients, while the association for women of fertile age was not univocal [97,98,118]. Furthermore, some authors have shown an inverse correlation between the circulating levels of APN and endometrial cancer staging, and women with lower APN levels, in fact, showed a more advanced endometrial cancer stage with a greater frequency [119].

The mechanisms by which APN inhibits the growth of endometrial cancer cells are not yet well known. However, several hypotheses have been formulated which imply the previously mentioned activation of AMPK (resulting in cell growth suppression and apoptosis), the extracellular signal-regulated protein kinase (ERK) and Akt pathway inhibition and the reduction of Cyclin D1 expression [120]. Furthermore, some authors have suggested that APN may reduce the Bcl2/Bax ratio, which causes an increase in the permeability of the mitochondrial membrane, resulting in the release of Cytochrome-C in the cell cytoplasm and, ultimately, in the activation of caspases-induced apoptosis [117]. Finally, the pro-apoptotic effect mediated by the APN seems to also occur in the endothelial cells of blood vessels, which makes it act as a strong anti-angiogenic factor [121].

Not only a lack of APN, but also a defect in its action seems to represent a negative prognostic factor that underlines the importance of this molecule in the prevention of the endometrial cancer [121]. Several authors have in fact shown that a lower expression of AdipoR1 in endometrial cancer cells is associated with more advanced tumor stages, a higher percentage of myometrial invasion and lymph node diffusion [117,120,121].

Summarizing, low circulating APN levels appear to be associated with an increased risk and worse prognosis of endometrial cancer (Table 1). APN might, therefore, represent a promising tool for the prevention and early diagnosis of this type of malignancy.

## 6. Ovarian Cancer

Ovarian cancer is the neoplasm burdened by the highest rate of lethality among female genital tract malignancies, and affects mainly peri- and postmenopausal women [122]. The main reason for this very high mortality rate is due to the fact that the majority of the cases of ovarian cancer are detected in advanced stages. About 80–90% of ovarian malignancies originate in cells of the ovarian epithelium, located on the surface of the gland. Other, less frequent histopathological phenotypes are stromal and germinal tumors, which develop from stromal tissue and germ cells, respectively [122].

The causes of ovarian cancer are not fully understood. The most important risk factors that have been identified are: genetic predisposition and history of ovarian neoplasia, Caucasian ethnicity, nulliparity, infertility, a high-fat diet and obesity [123].

Little evidence is available on the role of APN in ovarian cancer risk and progression. Some authors analyzed serum levels of APN and leptin in 52 patients with ovarian cancer, showing that both APN and leptin concentrations were significantly lower in women with ovarian cancer than in healthy individuals [101]. Other authors showed that women affected by ovarian cancer with a low leptin/adiponectin ratio had statistically longer progression-free survival times (using Kaplan–Meier survival estimates) than those with a higher leptin/adiponectin ratio [124]. The same trend was found in relation to the tumor responsiveness to chemotherapy, and women with a lower ratio in fact showed a better clinical response [125].

Furthermore, Li et al. showed that AdipoR1 expression levels in cancerous ovarian tissues represents an independent prognostic factor of the disease, being positively associated with overall survival in patients [126]. Finally, some authors showed that APN is able to repress human ovarian cancer cell growth and reverse the stimulatory effects of 17β-estradiol and IGF-1 on cell proliferation through the downregulation of their receptors [127]. In conclusion, although little available evidence has suggested a protective role of APN on ovarian carcinogenesis, additional studies are necessary to elucidate its function in ovarian tumor onset and progression.

## 7. Thyroid Cancer

Thyroid cancer is the most common endocrine malignancy [61]. The majority of lesions are represented by well-differentiated carcinomas, mainly papillary thyroid carcinoma and follicular carcinoma (85% and 12% of cases, respectively), while only a small part of thyroid neoplasms is represented by anaplastic carcinoma and medullary carcinoma [128]. The association between increased adiposity and the risk of thyroid cancer has not been univocally established. In a large meta-analysis, increase of weight, BMI, waist or hip circumference and waist-to-hip ratio were associated with a greater risk of papillary, follicular and anaplastic thyroid cancers [129]. Several hypotheses have been formulated to suggest potential mechanisms for this link, implicating factors such as inflammation, oxidative stress, hyperinsulinemia and a deregulated secretion of adipokines (mainly leptin and adiponectin) [130].

In 2011, Mitsiades et al. showed that patients with any form of thyroid cancer had significantly lower levels of circulating APN compared to healthy subjects [105]. These data were partially confirmed by a large, multicenter prospective study, which showed a relationship between low serum APN concentrations and the presence of thyroid cancer in female patients (but not male, probably due to the low percentage of males recruited for the study). The negative relationship with APN was, however, absent, even among women, when the time interval between blood collection and thyroid cancer diagnosis was less than six years [102]. Finally, in a recent paper, no direct association between decreased levels of APN and tumor size or stages was found [103].

Several in vitro studies have shown that even if thyroid cancer cells express both AdipoR1 and AdipoR2, papillary thyroid carcinoma cell lines express a significantly lower number of receptors than normal thyrocytes [105,131]. Finally, when APN levels were measured in patients with medullary carcinoma, no significantly different blood concentrations were found compared to controls [104]. Further studies are needed to elucidate the role of APN in thyroid cancer risk.

## 8. Prostate Cancer

Prostate cancer is the most common malignancy in males and represents the fifth leading cause of cancer death in men [61]. A sedentary lifestyle, together with a high-calorie and high-fat diet, are risk factors for the development of prostate cancer. Extensive evidence has shown that the excess adipose tissue is deeply involved in the onset and progression of prostate cancer [132,133]. Recent studies have highlighted the central role of APN and its receptors in prostate cancer, even if some evidence appears to be contradictory [134]. Immunohistochemistry analyses have shown a decreased expression of both AdipoR1 and AdipoR2 receptor isoforms in prostate neoplastic tissues in comparison to healthy prostate tissue [135]. Results of a meta-analysis indicated that concentrations of APN in prostate cancer patients were significantly lower than in controls [106]. Specifically, some authors showed that reduced concentrations of APN were related to prostate cancer development and progression [110]. Other authors showed that knockdown of APN leads to increased tumor proliferation and invasion, decreasing tumor suppressing genes [136]. A large prospective study on plasma APN levels and prostate cancer risk and survival showed that men with higher circulating APN concentrations had a decreased risk of developing poorly differentiated cancer or metastases [108]. Moreover, APN treatment has been demonstrated to increase cellular anti-oxidative protection and decrease oxidative stress in a dose-dependent manner in human prostate cancer cell lines [137]. Growing evidences indicate that APN performs an anti-proliferative action in prostate cancer cells, inhibiting dihydrotestosterone-activated cell proliferation [138]. The over-expression of APN in prostate cancer cell lines has been demonstrated to inhibit mTOR-mediated neoplastic cells proliferation [139]. Finally, Gao et al. showed that microRNA 323 is able to stimulate VEGF-A-mediated neo-angiogenesis in prostate cancer tissues through the downregulation of APN’s receptors [140].

In contrast with these results, several authors have indicated that there is no significant association between APN expression and prostate malignancy [109], or that there is even a significant positive correlation between APN concentrations and incidence of low or intermediate-risk prostate cancer [107]. Plasma APN levels were reported detectable at higher concentrations in subjects with T3 (advanced outside) than in subjects with T2 (confined within the prostate) stage cancer. Authors have notably suggested that cachexia during the final stages of prostate cancer could be a reason for this phenomenon [25]. Furthermore, some evidence has indicated that AdipoR2 expression is directly associated with prostate cancer progression and metastatization [141,142].

Several genetic polymorphisms can result in a predisposition to increased prostate cancer risk. In a metanalysis of 133 published studies, AdipoQ rs2241766 and AdipoR1 rs10920531 variants were related to a higher risk of prostate neoplasia. Conversely, AdipoR1 rs2232853 variant was associated with a lower risk of developing this type of malignancy [134]. Finally, three common AdipoQ polymorphisms were evaluated in a large cohort of patients with localized prostate cancer who underwent radical prostatectomy: AdipoQ rs182052 allele was associated with both a higher risk of biochemical recurrence and decreased APN levels. Stratified analyses showed that this correlation was more evident in patients with abdominal fat distribution [143].

In conclusion, according to the predominance of literature showing an inverse correlation between APN and the risk of prostate neoplasia, APN deficiency might be a potential biomarker for the early detection of prostate cancer. Elevating APN levels in prostate cancer patients could be, therefore, a useful therapeutic target. Nevertheless, considering that literature data appear to be sometimes conflicting, further studies, both epidemiological and experimental, are warranted to clarify the association between APN and the development of prostate neoplasia.

## 9. Adiponectin Role in Endocrine Cancer Metastasis

Metastasis is an extremely complex process that represents a major issue in the management of cancer. Besides its role in cancer promotion, aberrant APN secretion has also been associated with tumor spread and metastasis [144]. Regarding endocrine malignancies, APN has been demonstrated to be able to suppress many important processes related to metastatization such as adhesion, invasion and migration of breast cancer cells [29]. This may occur in an LKB1-mediated manner: APN increases the expression of LKB1, determining an increased phosphorylation of AMPK. This phenomenon is of crucial importance for the modulation of two tumor suppressors, TSC2 and TSC1, and leads to a decreased phosphorylation of p70S6 kinase (S6K) and, ultimately, to reduced cancer cell migration and invasion [144]. APN’s protective role on endocrine cancer metastasis is also partially regulated through the AMPK/Akt pathway. It has been in fact shown that APN-activated AMPK decreases the invasiveness of MDA-MB-231 cells by inducing PP2A-mediated Akt dephosphorylation [38].

APN was also demonstrated to have a suppressive effect on metastatic endometrial cancer cells [145]. APN was, in fact, able to inhibit leptin-induced cancer invasion, which requires the inactivation of the JAK/STAT3 pathway and the stimulation of AMPK signaling.

Future studies on the role of APN and other adipose tissue-secreting molecules on cancer invasion and metastatization should, therefore, focus also on LKB1-mediated effects, on the signaling pathways APN uses and on the potential interactions with other adipose tissue-secreting molecules that might contribute to the spreading of tumors.

## 10. Future Perspectives and Therapeutic Implications

Since many studies associate endocrine cancer protection with APN-mediated signaling, drugs intended to bypass APN by directly activating its molecular pathways have been investigated in order to find potential strategies (both prophylactic and therapeutic) and counteract tumor development [146]. However, efforts to engineer APN protein have often been challenging, partly due to a lack of knowledge on the peculiar actions of different APN isoforms [147]. Two suggestions have been proposed to possibly take advantage of the anti-cancer properties of APN: the identification/development of APN receptor agonists, and the increase of endogenous APN concentrations [16]. The first APN receptor agonist that was produced, ADP355, included several amino acids in its structure, which were able to stabilize the molecule protecting it from proteolytic enzymes. In vivo, intraperitoneal administration of 1 mg/kg/day ADP355 for 28 days in immunocompromised mice was demonstrated to suppress the development of human breast cancer xenografts by 31%, with a good safety profile [147]. It was also able to modulate different signaling pathways such as AMPK, STAT3, PIK3/Akt and ERK1/2 [147,148]. Using a high throughput assay, several naturally occurring APN receptor agonists were recently identified [149]. These compounds, acting preferably on AdipoR1 (e.g., matairesinol, arctiin, arctigenin, gramine) or AdipoR2 (e.g., syringin, parthenolide, taxifoliol, deoxyschizandrin) were demonstrated to share important anti-cancer properties with APN, including anti-proliferative and anti-inflammatory effects [149].

Furthermore, it is also conceivable to increase endogenous levels of APN. Peroxisome proliferator-activated receptor-gamma (PPARγ) ligands have been proposed as a promising tool to reach this therapeutic target. It has been demonstrated that thiazolidinediones (synthetic PPARγ ligands) are able to increase APN concentrations in a dose- and time-dependent manner [150]. Even if promising data have been initially produced on the anti-cancer role of several thiazolidinediones (such as troglitazone and efatutazone), their effects on tumors remain to be clarified, given that phase 2 trials failed to show sufficient efficacy [151,152]. Moreover, we must consider that modifying these receptor interactions could also result in unfavorable effects. In this regard, several possible side effects derived from chronic APN treatment (such as infertility, cardiac damage and reduced bone density) have been proposed by some authors [153,154]. For these reasons, further studies are needed to elucidate the clinical relevance of such therapeutic approaches. Finally, it must be remembered that APN may also be regulated by dietary or lifestyle habits. Daily consumption of fish, omega-3 and fiber supplements [83], together with aerobic exercise of moderate intensity, have in fact been demonstrated to significantly increase circulating APN concentrations [155].

## 11. Conclusions

Obesity represents a major health and social problem strongly increasing the risk for various severe complications comprising endocrine cancer development. Our understanding on obesity-associated malignancies has been rapidly improving during recent years. Among other mechanisms, growing interest has been placed on the regulation of adipocyte-secreted molecules as a critical factor influencing cancer pathophysiology.

APN has been recognized as a key mediator linking obesity and endocrine-related malignancies. The multifaceted role of APN includes a series of complex biological actions on different cancer metabolic pathways and tumor microenvironments. The majority of epidemiological evidence clearly demonstrates that hypoadiponectinemia is related to an increased risk of obesity-related malignancies and poor cancer prognosis. Nevertheless, APN has sometimes shown potentially contradicting actions in endocrine-related tumorigenesis. We believe that this topic represents a promising research field, but there remain several challenges before uniquely considering APN as a treatment strategy in cancer. A more profound understanding of the pathophysiological links between the different APN isoforms and endocrine-related malignancies is therefore required in order to develop effective and safe therapies. Moreover, molecular and environmental settings under which APN acts as an anti-inflammatory or pro-inflammatory adipokine need to be further examined. Finally, the specific roles of each APN receptor and signaling pathway on the different types of endocrine cancers also remain largely unknown. Further studies are therefore needed to clarify these aspects.

## Figures and Tables

**Table 1:** Epidemiological associations between APN and endocrine cancers according to available metanalyses or major case–control studies.

Endocrine Cancer	APN Association to Cancer	Year	# of Studies	Ref.
Breast cancer	Inverse association. Low serum APN levels are associated with breast cancer in pre- and postmenopausal Asian women.	2019	27	[81]
	Inverse association. APN is a biomarker of breast cancer risk in pre- and postmenopausal women, especially among Asians.	2018	31	[79]
	Inverse association. Low APN concentrations are associated with an increased risk of breast cancer.	2014	15	[71]
	Inverse association. Lower APN levels correlate with a higher risk of breast cancer in postmenopausal women.	2014	8	[73]
	Inverse association. High APN level might decrease the risk of postmenopausal breast cancer.	2013	17	[82]
Endometrial cancer	Inverse association. Low APN level increases the risk of endometrial cancer.	2016	18	[96]
	Inverse association. Higher APN levels might have a protective effect against endometrial cancer in postmenopausal women.	2015	12	[97]
	Inverse association. Higher serum APN concentrations are associated with a reduced risk of endometrial cancer, especially in postmenopausal women.	2015	12	[98]
	Inverse association. Each 1 µg/mL increase of APN level is associated with a 3% reduction in endometrial cancer risk.	2015	12	[99]
	Inverse association. Increased circulating APN and adiponectin/leptin ratio are associated with a decreased risk of endometrial cancer.	2015	13	[100]
Ovarian cancer	Inverse association. The mean APN concentrations in patients with ovarian cancer are lower than those of the control group.	2016	1	[101]
Thyroid cancer	Inverse association. APN is inversely associated with thyroid cancer risk among women, but not among men.	2018	1	[102]
	No association. No direct association between decreased levels of APN and papillary thyroid carcinoma size or stage was found.	2018	1	[103]
	No association. Serum APN levels are not significantly different between patients with or without medullary thyroid carcinomas.	2016	1	[104]
	Inverse association. Circulating APN is inversely associated with thyroid cancer risk.	2011	1	[105]
Prostate cancer	Inverse association. Decreased concentration of APN is associated with a greater risk of prostate cancer.	2015	11	[106]
	Direct association. The incidence of prostate cancer is increased in overweight men with high APN concentrations.	2015	1	[107]
	Inverse association. Higher APN levels reduce both the risk of developing high-grade prostate cancer and a risk of dying from the cancer.	2010	1	[108]
	Direct association. Serum APN levels are higher in advanced outside (relative to organ-confined) prostate cancers.	2008	1	[25]
	No association. APN was not of significantly associated with prostate cancer risk or high-grade disease.	2006	1	[109]
	Inverse association. APN levels are decreased in patients with prostate cancer and are also inversely associated with the histologic grade of the tumor.	2005	1	[110]

## Abbreviations

APN	Adiponectin
AdipoR1/2	Adiponectin receptor 1/2
BMI	Body mass index
TNF	Tumor necrosis factor
LMW	Low molecular weight
MMW	Middle molecular weight
HMW	High molecular weight
AMPK	Adenosine monophosphate-activated protein kinase
LKB1	Liver kinase B1
ATP	Adenosine triphosphate
mTOR	Mammalian target of rapamycin
JAK	Janus kinase
IL-6	Interleukin-6
STAT3	Signal transducer and activator of transcription 3
ERK1/2	Extracellular signal-regulated protein kinases 1 and 2
PP2A	Protein phosphatase 2A
ERα	Estrogen receptor alpha
VEGF	Vascular endothelial growth factor
CRP	C-reactive protein
MAPK	Mitogen-activated protein kinase
IGF-I	Insulin-like growth factor I
SHBG	Sex hormones binding globulin
PPARγ	Proliferator-activated receptor-gamma
S6K	p70S6 kinase

## References

[1] NCD Risk Factor Collaboration (NCD-RisC) (2016). Trends in adult body-mass index in 200 countries from 1975 to 2014: A pooled analysis of 1698 population-based measurement studies with 19.2 million participants. Lancet.

[2] Lu Y., Hajifathalian K., Ezzati M., Woodward M., Rimm E.B., Danaei G., Global Burden of Metabolic Risk Factors for Chronic Diseases Collaboration (BMI Mediated Effects) (2014). Metabolic mediators of the effects of body-mass index, overweight, and obesity on coronary heart disease and stroke: A pooled analysis of 97 prospective cohorts with 1.8 million participants. Lancet.

[3] Renehan A.G., Tyson M., Egger M., Heller R.F., Zwahlen M. (2008). Body-mass index and incidence of cancer: A systematic review and meta-analysis of prospective observational studies. Lancet.

[4] Khandekar M.J., Cohen P., Spiegelman B.M. (2011). Molecular mechanisms of cancer development in obesity. Nat. Rev. Cancer.

[5] Di Angelantonio E., Bhupathiraju Sh N., Wormser D., Gao P., Kaptoge S., Berrington de Gonzalez A., Cairns B.J., Huxley R., Jackson Ch L., Global BMI Mortality Collaboration (2016). Body-mass index and all-cause mortality: Individual-participant-data meta-analysis of 239 prospective studies in four continents. Lancet.

[6] Sciacca L., Vigneri R., Tumminia A., Frasca F., Squatrito S., Frittitta L., Vigneri P. (2013). Clinical and molecular mechanisms favoring cancer initiation and progression in diabetic patients. Nutr. Metab. Cardiov. Dis..

[7] Vigneri R., Goldfine I.D., Frittitta L. (2016). Insulin, insulin receptors, and cancer. J. Endocrinol. Invest..

[8] Vigneri P., Frasca F., Sciacca L., Frittitta L., Vigneri R. (2006). Obesity and cancer. Nutr. Metab. Cardiov. Dis..

[9] Booth A., Magnuson A., Fouts J., Foster M. (2015). Adipose tissue, obesity and adipokines: Role in cancer promotion. Hormone Mol. Biol. Clin. Invest..

[10] Scherer P.E. (2016). The Multifaceted Roles of Adipose Tissue-Therapeutic Targets for Diabetes and Beyond: The 2015 Banting Lecture. Diabetes.

[11] Di Zazzo E., Polito R., Bartollino S., Nigro E., Porcile C., Bianco A., Daniele A., Moncharmont B. (2019). Adiponectin as Link Factor between Adipose Tissue and Cancer. Int. J. Mol. Sci..

[12] Baratta R., Amato S., Degano C., Farina M.G., Patane G., Vigneri R., Frittitta L. (2004). Adiponectin relationship with lipid metabolism is independent of body fat mass: Evidence from both cross-sectional and intervention studies. J. Clin. Endocrinol. Metab..

[13] Patane G., Caporarello N., Marchetti P., Parrino C., Sudano D., Marselli L., Vigneri R., Frittitta L. (2013). Adiponectin increases glucose-induced insulin secretion through the activation of lipid oxidation. Acta Diabetol..

[14] Yamauchi T., Kamon J., Ito Y., Tsuchida A., Yokomizo T., Kita S., Sugiyama T., Miyagishi M., Hara K., Tsunoda M. (2003). Cloning of adiponectin receptors that mediate antidiabetic metabolic effects. Nature.

[15] Vansaun M.N. (2013). Molecular pathways: Adiponectin and leptin signaling in cancer. Clin. Cancer Res..

[16] Katira A., Tan P.H. (2016). Evolving role of adiponectin in cancer-controversies and update. Cancer Biol. Med..

[17] Menzaghi C., Trischitta V. (2018). The Adiponectin Paradox for All-Cause and Cardiovascular Mortality. Diabetes.

[18] Wong G.W., Wang J., Hug C., Tsao T.S., Lodish H.F. (2004). A family of Acrp30/adiponectin structural and functional paralogs. Proc. Natl. Acad. Sci. USA.

[19] Takemura Y., Ouchi N., Shibata R., Aprahamian T., Kirber M.T., Summer R.S., Kihara S., Walsh K. (2007). Adiponectin modulates inflammatory reactions via calreticulin receptor-dependent clearance of early apoptotic bodies. J. Clin. Invest..

[20] Yamauchi T., Iwabu M., Okada-Iwabu M., Kadowaki T. (2014). Adiponectin receptors: A review of their structure, function and how they work. Best Pract. Res. Clin. Endocrinol. Metab..

[21] Hug C., Wang J., Ahmad N.S., Bogan J.S., Tsao T.S., Lodish H.F. (2004). T-cadherin is a receptor for hexameric and high-molecular-weight forms of Acrp30/adiponectin. Proc. Natl. Acad. Sci. USA.

[22] Hebbard L., Ranscht B. (2014). Multifaceted roles of adiponectin in cancer. Best Pract. Res. Clin. Endocrinol. Metab..

[23] Dalamaga M., Diakopoulos K.N., Mantzoros C.S. (2012). The role of adiponectin in cancer: A review of current evidence. Endocr. Rev..

[24] Hefetz-Sela S., Scherer P.E. (2013). Adipocytes: Impact on tumor growth and potential sites for therapeutic intervention. Pharmacol. Ther..

[25] Housa D., Vernerova Z., Heracek J., Prochazka B., Cechak P., Kuncova J., Haluzik M. (2008). Adiponectin as a potential marker of prostate cancer progression: Studies in organ-confined and locally advanced prostate cancer. Physiol. Res..

[26] Izadi V., Farabad E., Azadbakht L. (2012). Serum adiponectin level and different kinds of cancer: A review of recent evidence. ISRN Oncol..

[27] Steinberg G.R., Kemp B.E. (2009). AMPK in Health and Disease. Physiol. Rev..

[28] Shackelford D.B., Shaw R.J. (2009). The LKB1-AMPK pathway: Metabolism and growth control in tumour suppression. Nat. Rev. Cancer.

[29] Taliaferro-Smith L., Nagalingam A., Zhong D., Zhou W., Saxena N.K., Sharma D. (2009). LKB1 is required for adiponectin-mediated modulation of AMPK-S6K axis and inhibition of migration and invasion of breast cancer cells. Oncogene.

[30] Manieri E., Herrera-Melle L., Mora A., Tomas-Loba A., Leiva-Vega L., Fernandez D.I., Rodriguez E., Moran L., Hernandez-Cosido L., Torres J.L. (2019). Adiponectin accounts for gender differences in hepatocellular carcinoma incidence. J. Exp. Med..

[31] Luo Z., Saha A.K., Xiang X., Ruderman N.B. (2005). AMPK, the metabolic syndrome and cancer. Trends Pharmacol. Sci..

[32] Li G., Cong L., Gasser J., Zhao J., Chen K., Li F. (2011). Mechanisms underlying the anti-proliferative actions of adiponectin in human breast cancer cells, MCF7-dependency on the cAMP/protein kinase-A pathway. Nutr. Cancer.

[33] Korner A., Pazaitou-Panayiotou K., Kelesidis T., Kelesidis I., Williams C.J., Kaprara A., Bullen J., Neuwirth A., Tseleni S., Mitsiades N. (2007). Total and high-molecular-weight adiponectin in breast cancer: In vitro and in vivo studies. J. Clin. Endocrinol. Metab..

[34] Zakikhani M., Dowling R.J., Sonenberg N., Pollak M.N. (2008). The effects of adiponectin and metformin on prostate and colon neoplasia involve activation of AMP-activated protein kinase. Cancer Prev. Res..

[35] Barb D., Neuwirth A., Mantzoros C.S., Balk S.P. (2007). Adiponectin signals in prostate cancer cells through Akt to activate the mammalian target of rapamycin pathway. Endocr. Relat. Cancer.

[36] Cong L., Gasser J., Zhao J., Yang B., Li F., Zhao A.Z. (2007). Human adiponectin inhibits cell growth and induces apoptosis in human endometrial carcinoma cells, HEC-1-A and RL95 2. Endocr. Relat. Cancer.

[37] Sablina A.A., Hahn W.C. (2007). The role of PP2A A subunits in tumor suppression. Cell Adhes. Migr..

[38] Kim K.Y., Baek A., Hwang J.E., Choi Y.A., Jeong J., Lee M.S., Cho D.H., Lim J.S., Kim K.I., Yang Y. (2009). Adiponectin-activated AMPK stimulates dephosphorylation of AKT through protein phosphatase 2A activation. Cancer Res..

[39] Bowman T., Garcia R., Turkson J., Jove R. (2000). STATs in oncogenesis. Oncogene.

[40] Park J., Euhus D.M., Scherer P.E. (2011). Paracrine and endocrine effects of adipose tissue on cancer development and progression. Endocr. Rev..

[41] Brown K.A., Simpson E.R. (2010). Obesity and breast cancer: Progress to understanding the relationship. Cancer Res..

[42] Stephens J.M., Elks C.M. (2017). Oncostatin M: Potential Implications for Malignancy and Metabolism. Curr. Pharm. Des..

[43] Pollak M. (2008). Insulin and insulin-like growth factor signalling in neoplasia. Nat. Rev. Cancer.

[44] Sciacca L., Vella V., Frittitta L., Tumminia A., Manzella L., Squatrito S., Belfiore A., Vigneri R. (2018). Long-acting insulin analogs and cancer. Nutr. Metab. Cardiov. Dis..

[45] Weyer C., Funahashi T., Tanaka S., Hotta K., Matsuzawa Y., Pratley R.E., Tataranni P.A. (2001). Hypoadiponectinemia in obesity and type 2 diabetes: Close association with insulin resistance and hyperinsulinemia. J. Clin. Endocrinol. Metab..

[46] Kelesidis I., Kelesidis T., Mantzoros C.S. (2006). Adiponectin and cancer: A systematic review. Br. J. Cancer.

[47] Sengupta S., Peterson T.R., Sabatini D.M. (2010). Regulation of the mTOR complex 1 pathway by nutrients, growth factors, and stress. Mol. Cell.

[48] Mantovani A., Marchesi F., Malesci A., Laghi L., Allavena P. (2017). Tumour-associated macrophages as treatment targets in oncology. Nat. Rev. Clin. Oncol..

[49] Stacker S.A., Williams S.P., Karnezis T., Shayan R., Fox S.B., Achen M.G. (2014). Lymphangiogenesis and lymphatic vessel remodelling in cancer. Nat. Rev. Cancer.

[50] Catalan V., Gomez-Ambrosi J., Rodriguez A., Fruhbeck G. (2013). Adipose tissue immunity and cancer. Front. Physiol..

[51] Mraz M., Haluzik M. (2014). The role of adipose tissue immune cells in obesity and low-grade inflammation. J. Endocrinol..

[52] Castoldi A., Naffah de Souza C., Camara N.O., Moraes-Vieira P.M. (2015). The Macrophage Switch in Obesity Development. Front. Immunol..

[53] Nigro E., Scudiero O., Monaco M.L., Palmieri A., Mazzarella G., Costagliola C., Bianco A., Daniele A. (2014). New insight into adiponectin role in obesity and obesity-related diseases. BioMed Res. Int..

[54] Yanai H., Yoshida H. (2019). Beneficial Effects of Adiponectin on Glucose and Lipid Metabolism and Atherosclerotic Progression: Mechanisms and Perspectives. Int. J. Mol. Sci..

[55] Yokota T., Oritani K., Takahashi I., Ishikawa J., Matsuyama A., Ouchi N., Kihara S., Funahashi T., Tenner A.J., Tomiyama Y. (2000). Adiponectin, a new member of the family of soluble defense collagens, negatively regulates the growth of myelomonocytic progenitors and the functions of macrophages. Blood.

[56] Hanahan D., Weinberg R.A. (2011). Hallmarks of cancer: The next generation. Cell.

[57] Brakenhielm E., Veitonmaki N., Cao R., Kihara S., Matsuzawa Y., Zhivotovsky B., Funahashi T., Cao Y. (2004). Adiponectin-induced antiangiogenesis and antitumor activity involve caspase-mediated endothelial cell apoptosis. Proc. Natl. Acad. Sci. USA.

[58] Bergers G., Hanahan D. (2008). Modes of resistance to anti-angiogenic therapy. Nat. Rev. Cancer.

[59] Mahadev K., Wu X., Donnelly S., Ouedraogo R., Eckhart A.D., Goldstein B.J. (2008). Adiponectin inhibits vascular endothelial growth factor-induced migration of human coronary artery endothelial cells. Cardiov. Res..

[60] Hebbard L.W., Garlatti M., Young L.J., Cardiff R.D., Oshima R.G., Ranscht B. (2008). T-cadherin supports angiogenesis and adiponectin association with the vasculature in a mouse mammary tumor model. Cancer Res..

[61] Bray F., Ferlay J., Soerjomataram I., Siegel R.L., Torre L.A., Jemal A. (2018). Global cancer statistics 2018: GLOBOCAN estimates of incidence and mortality worldwide for 36 cancers in 185 countries. Cancer J. Clin..

[62] Picon-Ruiz M., Morata-Tarifa C., Valle-Goffin J.J., Friedman E.R., Slingerland J.M. (2017). Obesity and adverse breast cancer risk and outcome: Mechanistic insights and strategies for intervention. Cancer J. Clin..

[63] Panis C., Herrera A., Aranome A.M.F., Victorino V.J., Michelleti P.L., Morimoto H.K., Cecchini A.L., Simao A.N.C., Cecchini R. (2014). Clinical insights from adiponectin analysis in breast cancer patients reveal its anti-inflammatory properties in non-obese women. Mol. Cell. Endocrinol..

[64] Carroll P.A., Healy L., Lysaght J., Boyle T., Reynolds J.V., Kennedy M.J., Pidgeon G., Connolly E.M. (2011). Influence of the metabolic syndrome on leptin and leptin receptor in breast cancer. Mol. Carcinog..

[65] Beck J.C., Hosick H.L., Watkins B.A. (1989). Growth of epithelium from a preneoplastic mammary outgrowth in response to mammary adipose tissue. In Vitro Cell. Dev. Biol..

[66] Iyengar P., Combs T.P., Shah S.J., Gouon-Evans V., Pollard J.W., Albanese C., Flanagan L., Tenniswood M.P., Guha C., Lisanti M.P. (2003). Adipocyte-secreted factors synergistically promote mammary tumorigenesis through induction of anti-apoptotic transcriptional programs and proto-oncogene stabilization. Oncogene.

[67] Dos Santos E., Benaitreau D., Dieudonne M.N., Leneveu M.C., Serazin V., Giudicelli Y., Pecquery R. (2008). Adiponectin mediates an antiproliferative response in human MDA-MB 231 breast cancer cells. Oncol. Rep..

[68] Arditi J.D., Venihaki M., Karalis K.P., Chrousos G.P. (2007). Antiproliferative effect of adiponectin on MCF7 breast cancer cells: A potential hormonal link between obesity and cancer. Hormone Metab. Res..

[69] Dieudonne M.N., Bussiere M., Dos Santos E., Leneveu M.C., Giudicelli Y., Pecquery R. (2006). Adiponectin mediates antiproliferative and apoptotic responses in human MCF7 breast cancer cells. Biochem. Biophys. Res. Commun..

[70] Miyoshi Y., Funahashi T., Kihara S., Taguchi T., Tamaki Y., Matsuzawa Y., Noguchi S. (2003). Association of serum adiponectin levels with breast cancer risk. Clin. Cancer Res..

[71] Macis D., Guerrieri-Gonzaga A., Gandini S. (2014). Circulating adiponectin and breast cancer risk: A systematic review and meta-analysis. In. J. Epidemiol..

[72] Tian Y.F., Chu C.H., Wu M.H., Chang C.L., Yang T., Chou Y.C., Hsu G.C., Yu C.P., Yu J.C., Sun C.A. (2007). Anthropometric measures, plasma adiponectin, and breast cancer risk. Endocr. Cancer.

[73] Ye J., Jia J., Dong S., Zhang C., Yu S., Li L., Mao C., Wang D., Chen J., Yuan G. (2014). Circulating adiponectin levels and the risk of breast cancer: A meta-analysis. Eur. J. Cancer Prev..

[74] Mantzoros C., Petridou E., Dessypris N., Chavelas C., Dalamaga M., Alexe D.M., Papadiamantis Y., Markopoulos C., Spanos E., Chrousos G. (2004). Adiponectin and breast cancer risk. J. Clin. Endocrinol. Metab..

[75] Tworoger S.S., Eliassen A.H., Kelesidis T., Colditz G.A., Willett W.C., Mantzoros C.S., Hankinson S.E. (2007). Plasma adiponectin concentrations and risk of incident breast cancer. J. Clin. Endocrinol. Metab..

[76] Cust A.E., Stocks T., Lukanova A., Lundin E., Hallmans G., Kaaks R., Jonsson H., Stattin P. (2009). The influence of overweight and insulin resistance on breast cancer risk and tumour stage at diagnosis: A prospective study. Breast Cancer Res. Treat..

[77] Hancke K., Grubeck D., Hauser N., Kreienberg R., Weiss J.M. (2010). Adipocyte fatty acid-binding protein as a novel prognostic factor in obese breast cancer patients. Breast Cancer Res. Treat..

[78] Macis D., Gandini S., Guerrieri-Gonzaga A., Johansson H., Magni P., Ruscica M., Lazzeroni M., Serrano D., Cazzaniga M., Mora S. (2012). Prognostic effect of circulating adiponectin in a randomized 2 x 2 trial of low-dose tamoxifen and fenretinide in premenopausal women at risk for breast cancer. J. Clin. Oncol..

[79] Gu L., Cao C., Fu J., Li Q., Li D.H., Chen M.Y. (2018). Serum adiponectin in breast cancer: A meta-analysis. Medicine.

[80] Jeong Y.J., Bong J.G., Park S.H., Choi J.H., Oh H.K. (2011). Expression of leptin, leptin receptor, adiponectin, and adiponectin receptor in ductal carcinoma in situ and invasive breast cancer. J. Breast Cancer.

[81] Yu Z., Tang S., Ma H., Duan H., Zeng Y. (2019). Association of serum adiponectin with breast cancer: A meta-analysis of 27 case-control studies. Medicine.

[82] Liu L.Y., Wang M., Ma Z.B., Yu L.X., Zhang Q., Gao D.Z., Wang F., Yu Z.G. (2013). The role of adiponectin in breast cancer: A meta-analysis. PLoS ONE.

[83] Silva F.M., de Almeida J.C., Feoli A.M. (2011). Effect of diet on adiponectin levels in blood. Nutr. Rev..

[84] Lyons A., Coleman M., Riis S., Favre C., O’Flanagan C.H., Zhdanov A.V., Papkovsky D.B., Hursting S.D., O’Connor R. (2017). Insulin-like growth factor 1 signaling is essential for mitochondrial biogenesis and mitophagy in cancer cells. J. Biol. Chem..

[85] Lai A., Sarcevic B., Prall O.W., Sutherland R.L. (2001). Insulin/insulin-like growth factor-I and estrogen cooperate to stimulate cyclin E-Cdk2 activation and cell Cycle progression in MCF-7 breast cancer cells through differential regulation of cyclin E and p21(WAF1/Cip1). J. Biol. Chem..

[86] Gavrila A., Chan J.L., Yiannakouris N., Kontogianni M., Miller L.C., Orlova C., Mantzoros C.S. (2003). Serum adiponectin levels are inversely associated with overall and central fat distribution but are not directly regulated by acute fasting or leptin administration in humans: Cross-sectional and interventional studies. J. Clin. Endocrinol. Metab..

[87] Georgiou G.P., Provatopoulou X., Kalogera E., Siasos G., Menenakos E., Zografos G.C., Gounaris A. (2016). Serum resistin is inversely related to breast cancer risk in premenopausal women. Breast.

[88] Guo M.M., Duan X.N., Cui S.D., Tian F.G., Cao X.C., Geng C.Z., Fan Z.M., Wang X., Wang S., Jiang H.C. (2015). Circulating High-Molecular-Weight (HMW) Adiponectin Level Is Related with Breast Cancer Risk Better than Total Adiponectin: A Case-Control Study. PLoS ONE.

[89] Wang Y., Lam J.B., Lam K.S., Liu J., Lam M.C., Hoo R.L., Wu D., Cooper G.J., Xu A. (2006). Adiponectin modulates the glycogen synthase kinase-3beta/beta-catenin signaling pathway and attenuates mammary tumorigenesis of MDA-MB-231 cells in nude mice. Cancer Res..

[90] Grossmann M.E., Nkhata K.J., Mizuno N.K., Ray A., Cleary M.P. (2008). Effects of adiponectin on breast cancer cell growth and signaling. Br. J. Cancer.

[91] Nakayama S., Miyoshi Y., Ishihara H., Noguchi S. (2008). Growth-inhibitory effect of adiponectin via adiponectin receptor 1 on human breast cancer cells through inhibition of S-phase entry without inducing apoptosis. Breast Cancer Res. Treat..

[92] Mauro L., Pellegrino M., De Amicis F., Ricchio E., Giordano F., Rizza P., Catalano S., Bonofiglio D., Sisci D., Panno M.L. (2014). Evidences that estrogen receptor alpha interferes with adiponectin effects on breast cancer cell growth. Cell Cycle.

[93] Panno M.L., Naimo G.D., Spina E., Ando S., Mauro L. (2016). Different molecular signaling sustaining adiponectin action in breast cancer. Curr. Opin. Pharmacol..

[94] Mauro L., Naimo G.D., Gelsomino L., Malivindi R., Bruno L., Pellegrino M., Tarallo R., Memoli D., Weisz A., Panno M.L. (2018). Uncoupling effects of estrogen receptor alpha on LKB1/AMPK interaction upon adiponectin exposure in breast cancer. FASEB J..

[95] Kang J.H., Lee Y.Y., Yu B.Y., Yang B.S., Cho K.H., Yoon D.K., Roh Y.K. (2005). Adiponectin induces growth arrest and apoptosis of MDA-MB-231 breast cancer cell. Archives Pharm. Res..

[96] Li Z.J., Yang X.L., Yao Y., Han W.Q., Li B.O. (2016). Circulating adiponectin levels and risk of endometrial cancer: Systematic review and meta-analysis. Exp. Ther. Med..

[97] Lin T., Zhao X., Kong W.M. (2015). Association between adiponectin levels and endometrial carcinoma risk: Evidence from a dose-response meta-analysis. BMJ open.

[98] Zeng F., Shi J., Long Y., Tian H., Li X., Zhao A.Z., Li R.F., Chen T. (2015). Adiponectin and Endometrial Cancer: A Systematic Review and Meta-Analysis. Cell. Physiol. Biochem..

[99] Zheng Q., Wu H., Cao J. (2015). Circulating adiponectin and risk of endometrial cancer. PLoS ONE.

[100] Gong T.T., Wu Q.J., Wang Y.L., Ma X.X. (2015). Circulating adiponectin, leptin and adiponectin-leptin ratio and endometrial cancer risk: Evidence from a meta-analysis of epidemiologic studies. Int. J. Cancer.

[101] Jin J.H., Kim H.J., Kim C.Y., Kim Y.H., Ju W., Kim S.C. (2016). Association of plasma adiponectin and leptin levels with the development and progression of ovarian cancer. Obstetrics Gynecol. Sci..

[102] Dossus L., Franceschi S., Biessy C., Navionis A.S., Travis R.C., Weiderpass E., Scalbert A., Romieu I., Tjonneland A., Olsen A. (2018). Adipokines and inflammation markers and risk of differentiated thyroid carcinoma: The EPIC study. Int. J. Cancer.

[103] Warakomski J., Romuk E., Jarzab B., Krajewska J., Sieminska L. (2018). Concentrations of Selected Adipokines, Interleukin-6, and Vitamin D in Patients with Papillary Thyroid Carcinoma in Respect to Thyroid Cancer Stages. Int. J. Endocrinol..

[104] Abooshahab R., Yaghmaei P., Ghadaksaz H.G., Hedayati M. (2016). Lack of Association between Serum Adiponectin/Leptin Levels and Medullary Thyroid Cancer. Asian Pac. J. Cancer Prev..

[105] Mitsiades N., Pazaitou-Panayiotou K., Aronis K.N., Moon H.S., Chamberland J.P., Liu X., Diakopoulos K.N., Kyttaris V., Panagiotou V., Mylvaganam G. (2011). Circulating adiponectin is inversely associated with risk of thyroid cancer: In vivo and in vitro studies. J. Clin. Endocrinol. Metab..

[106] Liao Q., Long C., Deng Z., Bi X., Hu J. (2015). The role of circulating adiponectin in prostate cancer: A meta-analysis. Int. J. Biol. Markers.

[107] Ikeda A., Nakagawa T., Kawai K., Onozawa M., Hayashi T., Matsushita Y., Tsutsumi M., Kojima T., Miyazaki J., Nishiyama H. (2015). Serum adiponectin concentration in 2,939 Japanese men undergoing screening for prostate cancer. Prostate Inte..

[108] Li H., Stampfer M.J., Mucci L., Rifai N., Qiu W., Kurth T., Ma J. (2010). A 25-year prospective study of plasma adiponectin and leptin concentrations and prostate cancer risk and survival. Clin. Chem..

[109] Baillargeon J., Platz E.A., Rose D.P., Pollock B.H., Ankerst D.P., Haffner S., Higgins B., Lokshin A., Troyer D., Hernandez J. (2006). Obesity, adipokines, and prostate cancer in a prospective population-based study. Cancer Epidemiol. Biomark. Prev..

[110] Goktas S., Yilmaz M.I., Caglar K., Sonmez A., Kilic S., Bedir S. (2005). Prostate cancer and adiponectin. Urology.

[111] Jemal A., Bray F., Center M.M., Ferlay J., Ward E., Forman D. (2011). Global cancer statistics. Cancer J. Clin..

[112] Orekoya O., Samson M.E., Trivedi T., Vyas S., Steck S.E. (2016). The Impact of Obesity on Surgical Outcome in Endometrial Cancer Patients: A Systematic Review. J. Gynecol. Surg..

[113] Crosbie E.J., Zwahlen M., Kitchener H.C., Egger M., Renehan A.G. (2010). Body mass index, hormone replacement therapy, and endometrial cancer risk: A meta-analysis. Cancer Epidemiol. Biomarkers Prev..

[114] Aarestrup J., Gamborg M., Ulrich L.G., Sorensen T.I., Baker J.L. (2016). Childhood body mass index and height and risk of histologic subtypes of endometrial cancer. Int. J. Obesity.

[115] van Kruijsdijk R.C., van der Wall E., Visseren F.L. (2009). Obesity and cancer: The role of dysfunctional adipose tissue. Cancer Epidemiol. Biomarkers Prev..

[116] Ma Y., Liu Z., Zhang Y., Lu B. (2013). Serum leptin, adiponectin and endometrial cancer risk in Chinese women. J. Gynecol. Oncol..

[117] Zhang L., Wen K., Han X., Liu R., Qu Q. (2015). Adiponectin mediates antiproliferative and apoptotic responses in endometrial carcinoma by the AdipoRs/AMPK pathway. Gynecol. Oncol..

[118] Cust A.E., Kaaks R., Friedenreich C., Bonnet F., Laville M., Lukanova A., Rinaldi S., Dossus L., Slimani N., Lundin E. (2007). Plasma adiponectin levels and endometrial cancer risk in pre- and postmenopausal women. J. Clin. Endocrinol. Metab..

[119] Rzepka-Gorska I., Bedner R., Cymbaluk-Ploska A., Chudecka-Glaz A. (2008). Serum adiponectin in relation to endometrial cancer and endometrial hyperplasia with atypia in obese women. Eur. J. Gynaecol. Oncol..

[120] Moon H.S., Chamberland J.P., Aronis K., Tseleni-Balafouta S., Mantzoros C.S. (2011). Direct role of adiponectin and adiponectin receptors in endometrial cancer: In vitro and ex vivo studies in humans. Mol. Cancer Ther..

[121] Yabushita H., Iwasaki K., Obayashi Y., Wakatsuki A. (2014). Clinicopathological roles of adiponectin and leptin receptors in endometrial carcinoma. Oncol. Lett..

[122] Romero I., Bast R.C. (2012). Minireview: Human ovarian cancer: Biology, current management, and paths to personalizing therapy. Endocrinology.

[123] Kalliala I., Markozannes G., Gunter M.J., Paraskevaidis E., Gabra H., Mitra A., Terzidou V., Bennett P., Martin-Hirsch P., Tsilidis K.K. (2017). Obesity and gynaecological and obstetric conditions: Umbrella review of the literature. BMJ.

[124] Diaz E.S., Karlan B.Y., Li A.J. (2013). Obesity-associated adipokines correlate with survival in epithelial ovarian cancer. Gynecol. Oncol..

[125] Slomian G.J., Nowak D., Buczkowska M., Glogowska-Gruszka A., Slomian S.P., Roczniak W., Janyga S., Nowak P. (2019). The role of adiponectin and leptin in the treatment of ovarian cancer patients. Endokr. Polska.

[126] Li X., Yu Z., Fang L., Liu F., Jiang K. (2017). Expression of Adiponectin Receptor-1 and Prognosis of Epithelial Ovarian Cancer Patients. Med. Sci. Monit..

[127] Hoffmann M., Gogola J., Ptak A. (2018). Adiponectin Reverses the Proliferative Effects of Estradiol and IGF-1 in Human Epithelial Ovarian Cancer Cells by Downregulating the Expression of Their Receptors. Hormones Cancer.

[128] Haugen B.R., Alexander E.K., Bible K.C., Doherty G.M., Mandel S.J., Nikiforov Y.E., Pacini F., Randolph G.W., Sawka A.M., Schlumberger M. (2016). 2015 American Thyroid Association Management Guidelines for Adult Patients with Thyroid Nodules and Differentiated Thyroid Cancer: The American Thyroid Association Guidelines Task Force on Thyroid Nodules and Differentiated Thyroid Cancer. Thyroid.

[129] Schmid D., Ricci C., Behrens G., Leitzmann M.F. (2015). Adiposity and risk of thyroid cancer: A systematic review and meta-analysis. Obesity Rev..

[130] Pappa T., Alevizaki M. (2014). Obesity and thyroid cancer: A clinical update. Thyroid.

[131] Cheng S.P., Liu C.L., Hsu Y.C., Chang Y.C., Huang S.Y., Lee J.J. (2013). Expression and biologic significance of adiponectin receptors in papillary thyroid carcinoma. Cell Biochem. Biophys..

[132] Hu M.B., Liu S.H., Jiang H.W., Bai P.D., Ding Q. (2014). Obesity affects the biopsy-mediated detection of prostate cancer, particularly high-grade prostate cancer: A dose-response meta-analysis of 29,464 patients. PLoS ONE.

[133] Hu M.B., Xu H., Bai P.D., Jiang H.W., Ding Q. (2014). Obesity has multifaceted impact on biochemical recurrence of prostate cancer: A dose-response meta-analysis of 36,927 patients. Med. Oncol..

[134] Hu M.B., Xu H., Hu J.M., Zhu W.H., Yang T., Jiang H.W., Ding Q. (2016). Genetic polymorphisms in leptin, adiponectin and their receptors affect risk and aggressiveness of prostate cancer: Evidence from a meta-analysis and pooled-review. Oncotarget.

[135] Michalakis K., Williams C.J., Mitsiades N., Blakeman J., Balafouta-Tselenis S., Giannopoulos A., Mantzoros C.S. (2007). Serum adiponectin concentrations and tissue expression of adiponectin receptors are reduced in patients with prostate cancer: A case control study. Cancer Epidemiol. Biomarkers Prev..

[136] Tan W., Wang L., Ma Q., Qi M., Lu N., Zhang L., Han B. (2015). Adiponectin as a potential tumor suppressor inhibiting epithelial-to-mesenchymal transition but frequently silenced in prostate cancer by promoter methylation. Prostate.

[137] Lu J.P., Hou Z.F., Duivenvoorden W.C., Whelan K., Honig A., Pinthus J.H. (2012). Adiponectin inhibits oxidative stress in human prostate carcinoma cells. Prostate Cancer Prostatic Dis..

[138] Bub J.D., Miyazaki T., Iwamoto Y. (2006). Adiponectin as a growth inhibitor in prostate cancer cells. Biochem. Biophys. Res. Commun..

[139] Gao Q., Zheng J., Yao X., Peng B. (2015). Adiponectin inhibits VEGF-A in prostate cancer cells. Tumour Biol..

[140] Gao Q., Yao X., Zheng J. (2015). MiR-323 Inhibits Prostate Cancer Vascularization Through Adiponectin Receptor. Cellular Physiol. Biochem..

[141] Rider J.R., Fiorentino M., Kelly R., Gerke T., Jordahl K., Sinnott J.A., Giovannucci E.L., Loda M., Mucci L.A., Finn S. (2015). Tumor expression of adiponectin receptor 2 and lethal prostate cancer. Carcinogenesis.

[142] Nguyen P.L., Ma J., Chavarro J.E., Freedman M.L., Lis R., Fedele G., Fiore C., Qiu W., Fiorentino M., Finn S. (2010). Fatty acid synthase polymorphisms, tumor expression, body mass index, prostate cancer risk, and survival. J.Clin. Oncol..

[143] Gu C., Qu Y., Zhang G., Sun L., Zhu Y., Ye D. (2015). A single nucleotide polymorphism in ADIPOQ predicts biochemical recurrence after radical prostatectomy in localized prostate cancer. Oncotarget.

[144] Saxena N.K., Sharma D. (2010). Metastasis suppression by adiponectin: LKB1 rises up to the challenge. Cell Adhes. Migr..

[145] Wu X., Yan Q., Zhang Z., Du G., Wan X. (2012). Acrp30 inhibits leptin-induced metastasis by downregulating the JAK/STAT3 pathway via AMPK activation in aggressive SPEC-2 endometrial cancer cells. Oncol. Rep..

[146] Hadad S.M., Fleming S., Thompson A.M. (2008). Targeting AMPK: A new therapeutic opportunity in breast cancer. Crit. Rev. Oncol. Hematol..

[147] Otvos L., Haspinger E., La Russa F., Maspero F., Graziano P., Kovalszky I., Lovas S., Nama K., Hoffmann R., Knappe D. (2011). Design and development of a peptide-based adiponectin receptor agonist for cancer treatment. BMC Biotech..

[148] Otvos L., Kovalszky I., Olah J., Coroniti R., Knappe D., Nollmann F.I., Hoffmann R., Wade J.D., Lovas S., Surmacz E. (2015). Optimization of adiponectin-derived peptides for inhibition of cancer cell growth and signaling. Biopolymers.

[149] Sun Y., Zang Z., Zhong L., Wu M., Su Q., Gao X., Zan W., Lin D., Zhao Y., Zhang Z. (2013). Identification of adiponectin receptor agonist utilizing a fluorescence polarization based high throughput assay. PLoS ONE.

[150] Maeda N., Takahashi M., Funahashi T., Kihara S., Nishizawa H., Kishida K., Nagaretani H., Matsuda M., Komuro R., Ouchi N. (2001). PPARgamma ligands increase expression and plasma concentrations of adiponectin, an adipose-derived protein. Diabetes.

[151] Burstein H.J., Demetri G.D., Mueller E., Sarraf P., Spiegelman B.M., Winer E.P. (2003). Use of the peroxisome proliferator-activated receptor (PPAR) gamma ligand troglitazone as treatment for refractory breast cancer: A phase II study. Breast Cancer Res. Treat..

[152] Williams R. (2015). Discontinued in 2013: Oncology drugs. Exp. Opin. Invest. Drugs.

[153] Ealey K.N., Kaludjerovic J., Archer M.C., Ward W.E. (2008). Adiponectin is a negative regulator of bone mineral and bone strength in growing mice. Exper. Biol. Med..

[154] Holland W.L., Scherer P.E. (2013). Cell Biology. Ronning after the adiponectin receptors. Science.

[155] Kriketos A.D., Gan S.K., Poynten A.M., Furler S.M., Chisholm D.J., Campbell L.V. (2004). Exercise increases adiponectin levels and insulin sensitivity in humans. Diabetes Care.

